# Heat shock protein 70 (HmHsp70) from *Hypsizygus marmoreus* confers thermotolerance to tobacco

**DOI:** 10.1186/s13568-020-0947-6

**Published:** 2020-01-18

**Authors:** Lili Xu, Jie Gao, Lizhong Guo, Hao Yu

**Affiliations:** Shandong Provincial Key Laboratory of Applied Mycology, College of Life Sciences, Qingdao Agricultural University, 700 Changcheng Road, Chengyang District, Qingdao, 266109 Shandong People’s Republic of China

**Keywords:** Heat shock protein 70, *Hypsizygus marmoreus*, Thermotolerance, Transgenic tobacco

## Abstract

The 70-kD heat shock proteins (Hsp70s) have been proved to be important for stress tolerance and protein folding and unfolding in almost all organisms. However, the functions of Hsp70s in mushroom are not well understood. In the present study, a *hsp70* gene from *Hypsizygus marmoreus*, *hmhsp70*, was cloned and transferred to tobacco (*Nicotiana tabacum*) to evaluate its function in thermotolerance. Sequence alignments and phylogenetic analysis revealed that HmHsp70 may be located in the mitochondria region. qPCR analysis revealed that the transcription level of *hmhsp70* in *H. marmoreus* mycelia increased after heat shock treatment in high temperature (42 °C) compared with untreated mycelia (at 25 °C). Transgenic tobaccos expressing *hmhsp70* gene showed enhanced resistance to lethal temperature compared with the wild type (WT) plants. Nearly 30% of the transgenic tobaccos survived after treated at a high temperature (50 °C and 52 °C for 4 h); however, almost all the WT tobaccos died after treated at 50 °C and no WT tobacco survived after heat shock at 52 °C. This study firstly showed the function of a *hsp70* gene from *H. marmoreus*.

## Introduction

*Hypsizygus marmoreus* (white var.) is one of the most popular edible mushrooms in East Asia (Wu et al. 2019; Mleczek et al. 2018). *H. marmoreus* is a low-temperature fruiting mushroom, the fruiting temperature is between 13 and 17 °C. The fruiting process of *H. marmoreus* is sensitive to temperature, and high temperature can lead to the death of mycelia or the malformation of the fruiting body (Qiu et al. 2013). Therefore, thermotolerance is important for *H. marmoreus* during both vegetative growth and fruiting process. Thermotolerance refers to the ability of an organism to cope with excessively high temperature, and it can be obtained by physiological change, morphological change or developmental change (Richter et al. 2010). The induced thermotolerance could also be acquired by either an exposure to short but sublethal high temperatures or by a gradual temperature increase to lethally high levels. For example, yeast experienced a sublethal temperature of 36 °C could acquire resistance to a lethal temperature of 52 °C (McAlister and Finkelstein 1980; Song et al. 2012; Wahid et al. 2007). Heat shock proteins (HSPs) are a group of conserved proteins that function as molecular chaperones and accumulate under heat shock. Previous studies suggest that HSPs proteins play a pivotal role in heat shock response (Kregel 2002; Richter et al. 2010).

HSPs are detected in all organisms when cells were exposed to various stresses such as heat, cold, heavy metal, or certain nutrients. HSPs are beneficial to cells helping them to adapt to the adverse environment. HSPs are classified based on molecular weight to Hsp100, Hsp90, Hsp70, Hsp60 and small HSPs (Kiang and Tsokos 1998). Of all HSPs, Hsp70 appears to correlate best with heat resistance either permanent or transient (Li and Mak 2009). Hsp70 family represents the most highly conserved of the HSPs and distributes in all organisms from prokaryotic and fungi to plants and animals (Li and Mak 2009; Richter et al. 2010; Xu et al. 2018). Hsp70s as molecular chaperone are involved in a wide variety of cellular processes, including protein biogenesis, protection of the proteome from stress, recovery of proteins from aggregates and facilitation of protein translocation across membranes (Clerico et al. 2015, 2019; Kiang and Tsokos 1998; Sekhar et al. 2018). Overexpression of Hsp70 coding genes could confer thermotolerance to organisms. The inducible expression of Hsp70 has been observed in lots of species, such as *Arabidopsis*, *Drosophila*, eubacteria and yeast (Gong and Golic 2006; Nwaka et al. 1996; Schumann 2007; Su and Li 2008). Mutations with *hsp70* deletions reduced thermotolerance in these organisms, and the thermotolerance defect could be complemented by expression *hsp70* genes. These studies proved that Hsp70 is essential for organisms to survive a severe heat shock. Besides, thermotolerance could not only be given by the overexpression of its own *hsp70* genes but also by transgenic *hsp70* genes from other organisms. The high conservation of Hsp70 gives its functional properties across the species. Overexpression of herbaceous peony Hsp70, PlHSP70, confers *A. thaliana* high temperature tolerance (Zhao et al. 2019). Transgenic *Arabidopsis* expressing the *hsp70* gene from *T. harzianum* T34 exhibited enhanced tolerance to heat, oxidative, osmotic and salt stresses (Montero-Barrientos et al. 2010). Therefore, it is a feasible way to study the function of Hsp70 in model organism.

In fungi, Hsp70 proteins perform chaperone dependent or independent functions and play a major role in various stress conditions (Tiwari et al. 2015). Fourteen genes of the Hsp70 family have been characterized in yeast and at least five of them are heat shock inducible (Boorstein and Craig 1990; Craig and Jacobsen 1984; Miura et al. 2006; Werner-Washburne and Craig 1989; Young and Craig 1993). Mutation or afunction of these *hsp70* genes would cause a temperature sensitive growth (Craig and Jacobsen 1984; Nelson et al. 1992). The role of Hsp70s in heat stress response in filamentous fungi has been studied for many years (Britton and Kapoor 2002; Caruso et al. 1987; Montero-Barrientos et al. 2008). Higher transcription level was observed when two pathogenic fungi, *Histoplama capsulatum* G222B *and H. capsulatum* Downs, were shocked at 34 °C or 37 °C. The strain, G222B, with higher thermotolerance has higher *hsp70* transcription level (Caruso et al. 1987). Overexpression of *hsp70* gene in *Trichoderma harzianum* T34 gave rise to transformants with high quantities of biomass obtained after heat shock treatment (Montero-Barrientos et al. 2008). Recently, some studies have related these proteins with heat stress resistance in edible mushrooms. The expression of *hsp70* genes in *Lentinula edodes* and *Pleurotus ostreatus* were upregulated under heat stress (Fu et al. 2016; Wang et al. 2018a; Zou et al. 2018), and the Hsp70s were more highly expressed in heat-tolerance strain than heat-sensitive strain. Even so, functional studies of *hsp70* genes are quite limited in mushroom compared to yeast and filamentous fungi.

In the previous study, we analyzed the global protein expression of *H. marmoreus* mycelia under optimal temperature (25 °C) and high temperature (42 °C) (Liu et al. 2014) by two-dimensional polyacrylamide gel electrophoresis. Seven highly expressed proteins, including a Hsp70 protein, were identified by MALDI-TOF-MS analysis, and these proteins were considered to provide the heat resistance to *H. marmoreus*. Based on these results, in this study, we cloned the Hsp70 protein coding gene, *hmhsp70*, from *H. marmoreus* and transformed tobacco with *hmhsp70* gene to determine the role of HmHsp70 in heat-shock response of plants. Our data indicated that HmHsp70 could give thermotolerance to tobacco.

## Materials and methods

### Strain and plant material and cultivation conditions

*Hypsizygus marmoreus* G12 provided by Shandong Provincial Key Laboratory of Applied Mycology was used in this study. The wild type (WT) tobacco (*Nicotiana tabacum* var. Xanthinc) was provided by Key Lab of Plant Biotechnology in Universities of Shandong Province. *Escherichia coli* Trans1-T1 (Transgen, Beijing, China) was used as host for plasmid construction and propagation. *Agrobacterium tumefaciens* LBA4404 was used as the T-DNA donor for tobacco transformation. *H. marmoreus* mycelia were cultivated in potato dextrose agar (PDA: extract from 200 g potato, 20 g dextrose, 20 g agar) media in 90 mm diameter petri dishes sealed with plastic wrap. The plates were incubated in a growth chamber at 25 ± 1 °C in darkness. For subculture, mycelial plugs (8 mm diameter) were cut from the margin of old agar cultures and placed in the center of the new media. WT tobacco was germinated in 1/2 Murashige and Skoog (MS) medium in a growth chamber at 25/22 °C (day/night) under a 16/8 h (day/night) photoperiod with illumination intensity of 3000 Lx. *E. coli* cells were grown in Luria-Bertani (LB) broth or on LB plates supplemented with kanamycin (Kan, 50 μg/mL) when required. *A. tumefaciens* cells were grown in minimal media (10 mM K_2_HPO_4_, 10 mM KH_2_PO_4_, 2.5 mM NaCl, 2 mM MgSO_4_·7H_2_O, 0.7 mM CaCl_2_, 9 μM FeSO_4_·7H_2_O, 4 mM (NH_4_)_2_SO_4_, 10 mM glucose, pH 7.0).

### Cloning the full-length cDNA of *hmhsp70* gene

The genome of *H. marmoreus* mycelia was extracted using EZNA Fungal DNA Mini Kit (Omega Bio-Tek, USA) according to the manufacture’s instruction. *H. marmoreus* mycelia were treated at 42 °C for 1 h, and then the total RNA was extracted from the treated mycelia frozen in liquid nitrogen by Trizol reagent method (RNAiso Plus, Takara, Japan) according to the manufacturer’s protocol. cDNA library was prepared using RNA PCR Kit (AMV) Ver.3.0 (Takara, Japan) according to the manufacture’s instruction. The DNA fragment of *hmhsp70* gene was amplified using degenerate primers De-F (5′-GCTGTHRTYACHGTYCCAGCTTAYTTC-3′)/De-R (5′-RGCDACRGCYTCRTCNGGRTTRAT-3′) with *H. marmoreus* genomic DNA as the template. The PCR product was sequenced using Sanger sequencing method in Sangon Biotech (Shanghai, China) Co. The 3′ end and 5′ end of the *hmhsp70* mRNA was confirmed using 3′ RACE and 5′ RACE methods using SMART TM RACE cDNA Amplification Kit (Clontech, CA, USA) according to the manufacture’s instruction. The whole cDNA fragment of *hmhsp70* gene was amplified using primers HSPorf-F (5′-GGGGACCTTTCTCTCTATT-3′) and HSPorf-R (5′-GATTTGTCATGCATGTGAG-3′) using the product of 5′ RACE as the template, and cloned into pMD18-T plasmid (Takara, Japan) according to the manufacture’s instruction. The sequence of inserted DNA fragment was sequenced by Sanger sequence in Sangon Biotech (Shanghai, China) Co.

### Construct the recombinant plasmid and plant transformation

The plant transformation vector pROK2 was used for tobacco transformation. The *hmhsp70* gene was cloned with primers ROK-*hsp*-F (5′-TGCTCTAGACTCTATTCCCTACCATCATGTTC-3′, the XbaI restriction site is underlined) and ROK-*hsp*-R (5′-CGGGGTACCTTGCGACTAACAAATATGCT-3′, the KpnI restriction site is underlined) using cDNA as the template. The *hmhsp70* gene fragment was purified by gel purification kit (Sangon, Shanghai, China) and cut with KpnI and XbaI. The restriction fragment was purified and cloned into pROK2 vector between KpnI and XbaI sites under the control of the CaMV 35S promoter to produce pROK2-*hsp* (Baulcombe et al. 1986). The hygromycin (Hyg) resistance gene, *hyg*, was amplified from pCAMBIA1301 using primers ROK-*hyg*-F (5′-CCCAAGCTTTCACAATTCCACACAACATAC-3′, the HindIII restriction site is underlined) and ROK-*hyg*-R (5′-CGGCTAGCTCTGGATTTTAGTACTGGATT-3′, the NheI restriction site is underlined). The PCR product was purified and cut with HindIII and NheI. Then the *hyg* restriction fragment was cloned into pROK2-*hsp* (cut with the same restriction enzymes) to produce pROK2-*hyghsp*.

The recombinant plasmid, pROK2-*hyghsp*, was introduced into *A. tumefaciens* LBA4404 by freeze-thaw method (Hofgen and Willmitzer 1988). Transformation of tobacco leaf disc was performed using an *Agrobacterium* mediated transformation method as described by Horsch et al. (Horsch et al. 1985; Voelker et al. 1987). Tobacco transformants were selected on MS medium supplemented with 0.5 μg/mL 6-benzylaminopurine, 400 μg/mL cefotaxime and 60 μg/mL Kan for 2 d and then they were transferred into selection MS medium plate containing 150 μg/mL Kan and 25 mg/L Hyg. Adventitious shoots were transferred to rooting medium supplemented with 0.5 μg/mL 1-naphthylacetic acid and 20 mg/L sucrose. Then, the transgenic and WT tobaccos were transferred into tissue cultivation bottles and grown in a controlled environmental growth chamber under the conditions described above.

### Thermotolerance assay

Tobacco of the WT and transgenic type were used for the thermotolerance assay using a growth chamber. WT and transgenic tobacco plants were shocked at 46 °C, 48 °C, 50 °C and 52 °C for 4 h, then further incubated at 25 °C for a week. Thermotolerance was evaluated based on survival number.

### Real-time quantitative PCR (qPCR) analysis of expression of *hmhsp70* gene

*H. marmoreus* mycelia on PDA plate were exposed at 42 °C for 0.5 h, 1 h, 2 h, 3 h and 4 h, respectively. Then the total RNA was isolated from mycelia immediately with Trizol reagent (RNAiso Plus, Takara), respectively. cDNA was synthesized as mentioned above. qPCR was performed using an Optical 96-well Fast Thermal Cycling Plate with the ABI 7500 real-time PCR system (Applied Biosystems, San Francisco). The reaction system was prepared using 2× SYBR Green SuperReal PreMix Plus (TianGen, Beijing, China) according to the manufacture’s instruction in a total volume of 15 μL for each tube. The thermal cycle used was 95 °C for 2 min, then 40 cycles of 10 s at 95 °C, 40 s at 60 °C. The 18S rRNA gene was used as internal reference gene. To detect the expression of *hmhsp70* genes in transgenic tobaccos, the total RNA was extracted from the leave of the transgenic tobaccos. qPCR reaction was used to detect the expression of *hmhsp70* gene, and the *actin* gene was used as internal reference gene. Primers used in qPCR reactions are listed in Table 1. Relative expression levels were calculated using the relative 2^−(*ΔΔCt*)^ method, and the expression of *hmhsp70* was normalized to the expression level of the 18S rRNA or *actin* gene.

**Table 1:** Primers used for real-time qPCR analysis

Primer name	Sequence (5′–3′)
Q-*actin*-F	CCTGAGGTCCTTTTCCAACCA
Q-*actin*-R	GGATTCCGGCAGCTTCCATT
Q-*18S*-F	GAGGGACCTGAGAAACG
Q-*18S*-R	ATAAGACCCGAAAGAGCC
Q-*hmhsp70*-F	GCTCACTCATTGCGCGGAATA
Q-*hmhsp70*-R	CCGACAAGACGTTCACCATGC

### Data analysis

Multiple sequence alignment of protein sequences was performed by Clustal X algorithm (Larkin et al. 2007). Phylogenetic tree analysis was performed with Mega 6.0 software (Tamura et al. 2013).

### Nucleotide sequence accession numbers

The 2001 bp nucleotide sequence of *hmhsp70* gene has been deposited in the GenBank DNA database under accession number of GQ246176.

## Results

### Clone and sequence analysis of HmHsp70 from *H. marmoreus*

Partial sequence of *hsp70* gene was amplified from cDNA library of heat treated *H. marmoreus* using degenerate primers. The primers were designed according to conserved sequences of heat shock gene from *Achlya klebsiana* (AKU02504), *Candida glabrata* (AY077689), *Rhizopus stolonifer* (AY147869). The cDNA ends of *hsp70* gene were confirmed by 3′ RACE and 5′ RACE methods. A 2308 bp DNA fragment, which contains a 2001 bp coding sequence, was obtained and designated as *hmhsp70* gene (GQ246176). The *hmhsp70* gene contains 10 exons and 9 introns. The deduced HmHsp70 protein contains 667 amino acids and the calculated molecular weight of HmHsp70 is 72.2 kDa. This is the first *hsp70* gene cloned from *H. marmoreus*, which may be helpful to uncover the underlying molecular mechanism of thermotolerance in *H. marmoreus*.

**Fig. 1:**
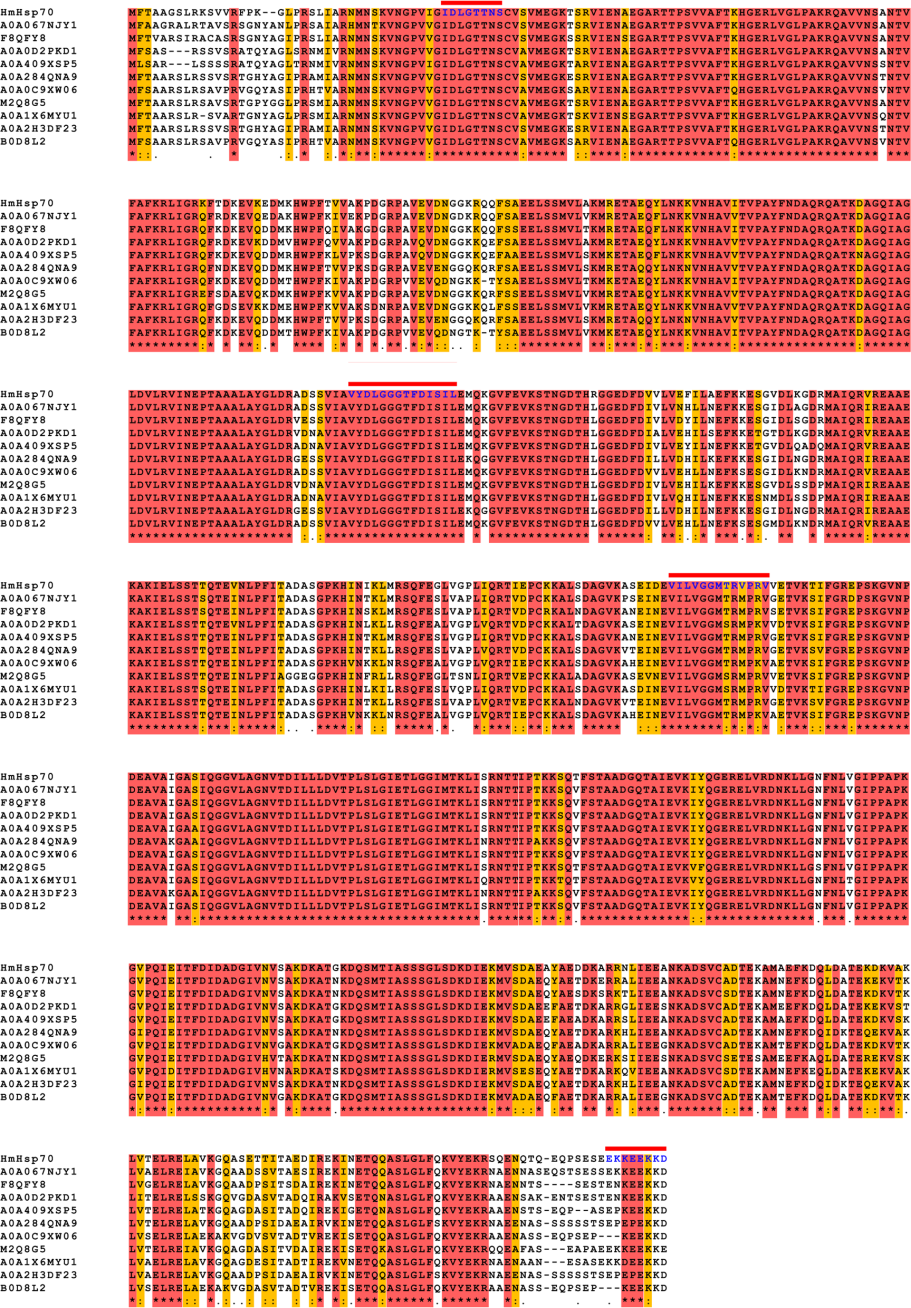
Alignments of the deduced amino acid sequences of HmHsp70 and 10 relative Hsp70 proteins from basidiomycetes fungi. Hsp70 family signature sequences are indicated with bars above the sequences

**Fig. 2:**
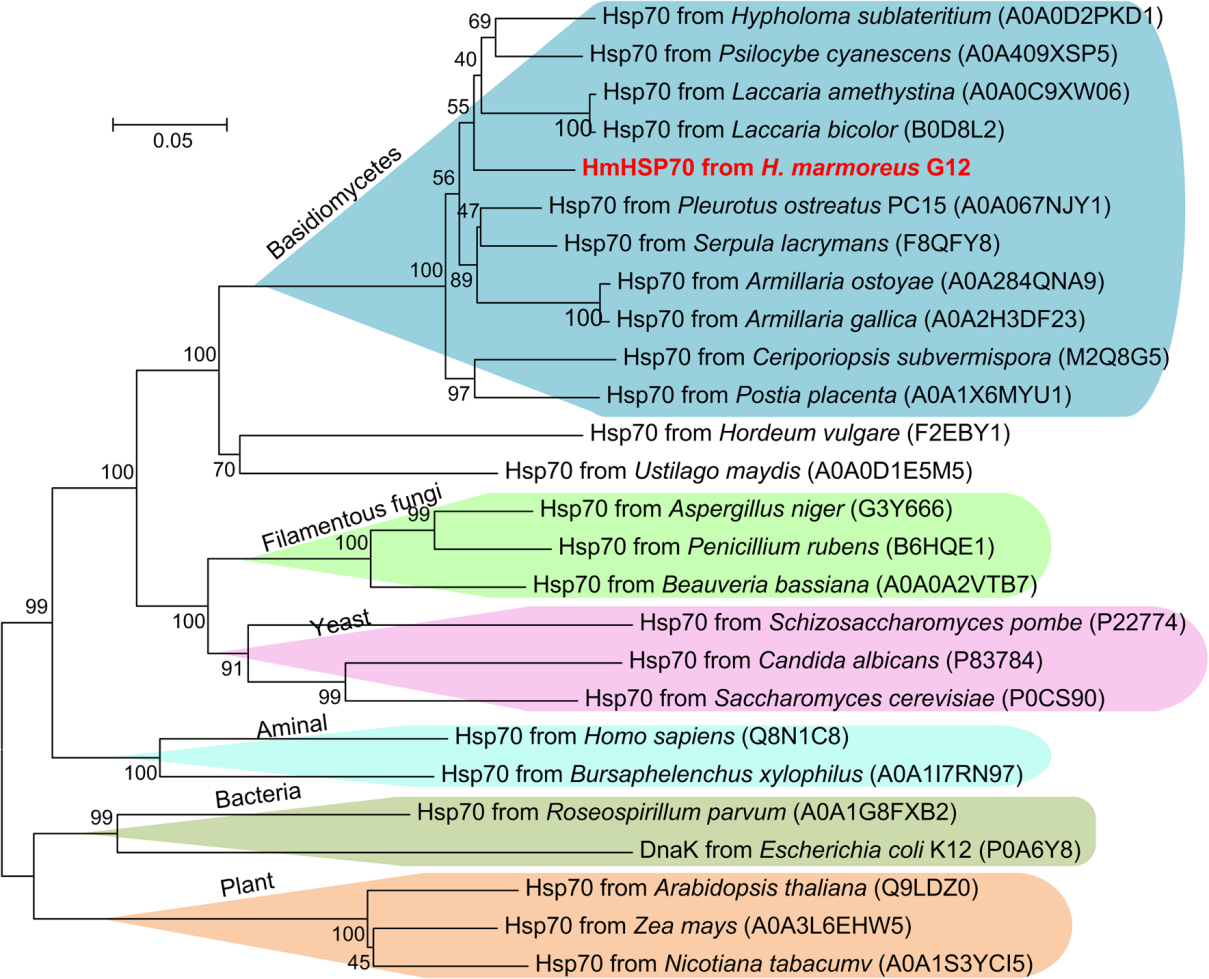
A neighbor-joining phylogenetic tree of HmHsp70 and relative Hsp70 proteins made by MEGA 6.0 software. GenBank accession numbers are indicated in parentheses. Numbers next to nodes indicate bootstrap values from 1000 replicates. Bar indicates evolutionary distance of 0.05 per 1000 amino acid positions

A BLAST search in GenBank database was performed and revealed that HmHsp70 of *H. marmoreus* shared high homology with HSPs in other basidiomycetes fungi such as *Pleurotus ostreatus*, *Hypholoma sublateritium* and *Psilocybe cyanescens*. Three Hsp70 family signatures were found in HmHsp70 sequence. The first signature (IDLGTTNS) was found at positions 39–46 in the *N*-terminal section, and the others were located at positions 227–240 (VYDLGGGTFDISIL) and positions 368–380 (VILVGGMTRVPRV) in the central part of HmHsp70 (Fig. 1). To investigate the evolutionary relationship of HmHsp70 and other Hsp70 proteins, a neighbor-joining tree was constructed after the alignment of Hsp70 proteins from different organisms: HmHsp70, ten Hsp70 proteins with highest sequence identity to HmHsp70 in UniProt database, and Hsp70 proteins with highest sequence identity to HmHsp70 from human, plant, bacteria, nematoda, tobacco, maize, *Arabidopsis*, *Saccharomyces*, fission yeast, *Aspergillus*, *Penicillium*, *Beauveria* and *Ustilago*, respectively (Fig. 2). HmHsp70 is associated with Hsp70 proteins from basidiomycetes fungi, and exhibits the highest sequence identity (88.7%) with Hsp70 from *P. ostreatus* PC15. It could be speculated that Hsp70s from basidiomycetes fungi evolved from the same ancestral HSP protein. Furthermore, the Hsp70 proteins from plants, animal, yeast, filamentous fungi and bacteria are clustered, respectively. The results indicated that Hsp70 proteins are highly conserved and could be used as molecular scale in biological classification.

### Expression of *hmhsp70* gene in *H. marmoreus*

**Fig. 3:**
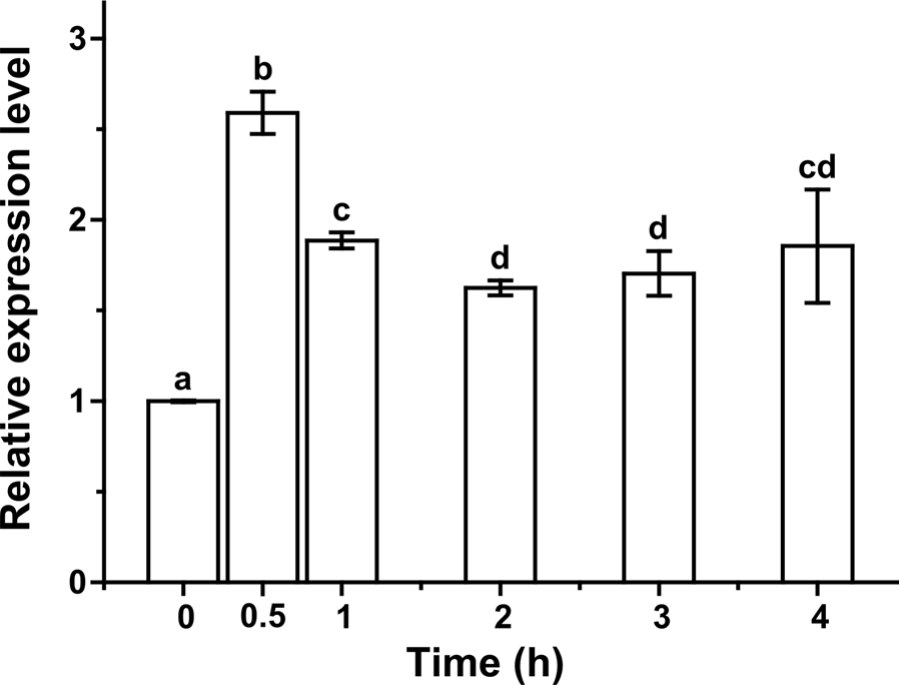
Expression profile of *hmhsp70* under heat stress. Mycelia of *H. marmoreus* were treated at 42 °C for 0, 0.5, 1, 2, 3 and 4 h. The 18S rRNA gene was used as an internal control. Each value is the mean from three parallel replicates ± SD. One-tailed *t* test, performed by SPSS 18.0 (Chicago, Illinois), was used to calculate statistical significance. Different letters indicate a significant difference at P < 0.05

The expression pattern of *hmhsp70* gene in *H. marmoreus* was investigated by qPCR analysis. *H. marmoreus* mycelia were exposed to a high temperature of 42 °C for 0 h, 0.5 h, 1 h, 2 h, 3 h and 4 h, respectively. After heat stress treatment, total RNA was isolated from mycelia. cDNA library was synthesized from RNA and used as the template for qPCR analysis. The expression of *hmhsp70* was significantly increased after heat shock at 42 °C (Fig. 3). The expression level was rapidly triggered and peaked at 0.5 h with a 1.48-fold increase (Fig. 3). After 0.5 h, the expression of *hmhsp70* gradually decreased. The expression level of *hmhsp70* still remained at a significant high level at 4 h compared to untreated mycelia. The results of qPCR confirmed the involvement of *hmhsp70* in heat shock response in *H. marmoreus*.

### Transformation and constitutive expression of *hmhsp70* gene in tobacco

**Fig. 4:**
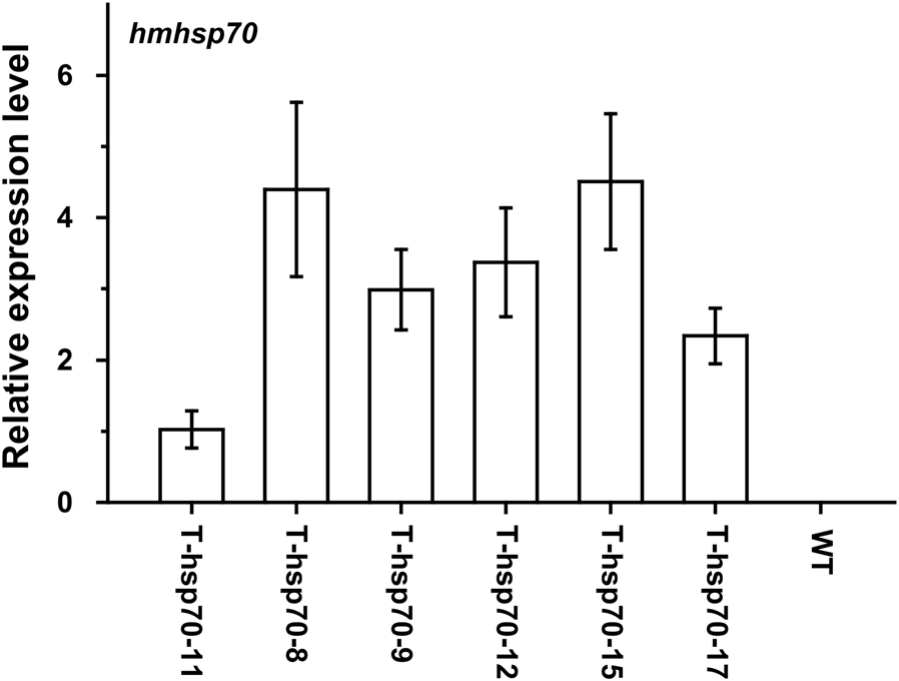
Expression of *hmhsp70* gene in transgenic tobaccos. The 2^−(*ΔΔCt*)^ values normalized to *actin* gene were averaged for each investigated transgenic tobaccos. The expression level of T-hsp70-11 was considered to be the reference expression level

To clarify the role of *hmhsp70* gene in thermotolerance, we introduced *hmhsp70* gene into tobacco. Tobacco is an idea platform to test the effect of stress response proteins not only from plants but also from other species (Sanmiya et al. 2004; Zhu et al. 2018). The full length of *hmhsp70* gene was inserted into plasmid pROK2 under the control of CaMV 35S promoter, and the recombinant plasmid was transformed into WT tobaccos by *Agrobacterium* mediated transformation. Twelve independent transgenic tobaccos (T0) were generated. Transformation of *hmhsp70* gene in these plants was confirmed by PCR using the genomic DNA from transgenic tobaccos as the templates. A clear band at about 2000 bp for transgenic tobacco was selected as positive transformants, while the negative WT tobacco control did not show any band. In addition, qPCR analysis was used to detect the expression level of *hmhsp*70 in transgenic tobaccos. qPCR results indicated that *hmhsp70* gene was introduced into the transgenic tobacco and successfully expressed in transgenic tobaccos (Fig. 4). Three transgenic tobacco lines, T-hsp70-8, T-hsp70-12 and T-hsp70-15, with high *hmhsp70* expression level were selected for the following experiments.

### HmHsp70 confers thermotolerance to tobacco

**Fig. 5:**
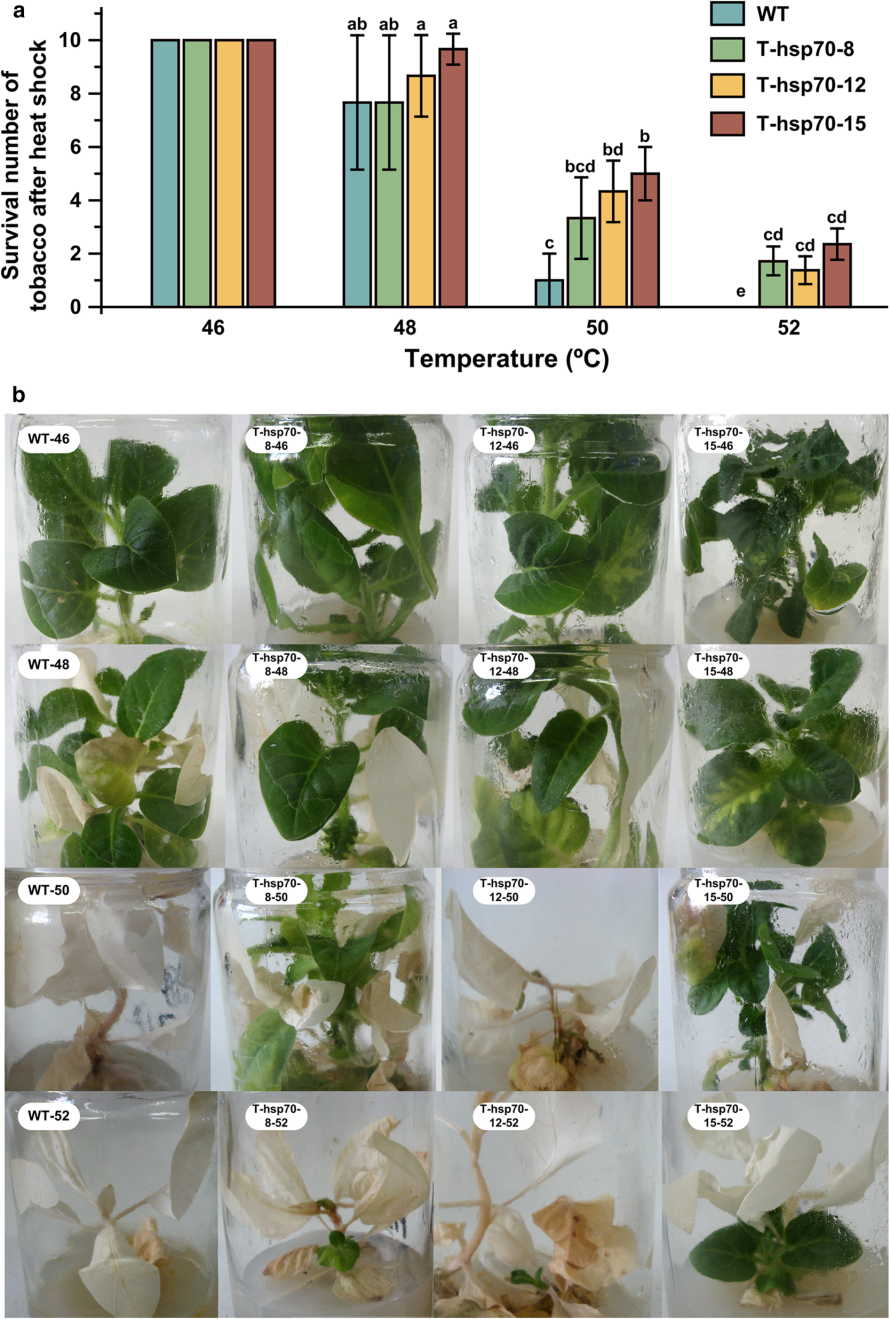
Enhanced heat tolerance of *hmhsp70* overexpression transgenic tobaccos. **a** The survival numbers of heat-shocked WT and transgenic tobaccos after 7 days of recovery growth at 25 °C. The transgenic and WT tobaccos grew on normal temperature (25 °C) in tissue culture bottles were shifted to high temperature (46 °C, 48 °C, 50 °C, 52 °C) for 4 h. **b** The typical phenotype of heat-shocked WT and transgenic tobaccos after 7 days of recovery growth at 25 °C. The shock temperature was indicated in the photos. Each value is the mean from three parallel replicates ± SD. Two-tailed *t* test, performed by SPSS 18.0 (Chicago, Illinois, USA), was used to calculate statistical significance. Different letters in the same group of bars indicate a significant difference (P < 0.05)

The growth of transgenic tobaccos and WT tobaccos has no difference under normal temperature (25 °C) (Fig. 5). In order to confirm the ability of *hmhsp70* overexpression tobaccos to heat, WT and transgenic tobaccos were treated at different temperature 46 °C, 48 °C, 50 °C, 52 °C for 4 h. Then the temperature was changed back to 25 °C and the heat treated tobaccos grew for 7 days. Ten tobaccos of each group were treated at one time, and the experiments repeated three times. The survival number of the heat treated tobaccos was recorded and compared. All the WT and transgenic tobaccos survived after heat shock at 46 °C, and no significant different survival number was observed after treated at 48 °C(Fig. 5a). The survival numbers of transgenic tobaccos are significantly higher than WT tobaccos after heat shock at 50 °C and 52 °C (Fig. 5a). Most of the WT tobaccos are died after treated at 50 °C and all WT tobaccos died after treated at 52 °C; however, 2 to 6 of the transgenic tobaccos survived. Old leaves are more sensitive to heat shock and new leaves grow from the stalk (Fig. 5b). The results indicated that transgenic tobaccos showed more heat tolerance than WT tobaccos in high lethal temperature.

## Discussions

Previous studies on *H. marmoreus* have mainly focused on cultivation, nutrition and chemical compounds (Liu et al. 2014; Mleczek et al. 2018; Qiu et al. 2013), but little is known about stress resistance of *H. marmoreus*. Heat shock is an important adverse environmental stress that influences the growth and development of mushrooms. Therefore, understanding of physiological alterations in response to heat stress and the corresponding mechanisms involved is essential for the breeding of heat-resistance *H. marmoreus* strains (Liu et al. 2019). Although, the heat shock response has been studied in considerable detail in yeast and plant (McAlister and Finkelstein 1980; Richter et al. 2010; Song et al. 2012), the mechanism of heat shock response in basidiomycetes remains elusive. Mushrooms evolved various strategies to response and alleviate heat shock stress, including HSPs synthesis, trehalose accumulation, and reactive oxygen species scavenging (Chen et al. 2017; Liu et al. 2016, 2018, 2019; Wang et al. 2017). In this study, we focused on a HSPs gene involved in heat shock response in *H. marmoreus*. A new *hsp70* gene was isolated from *H. marmoreus* and characterized. HmHsp70 exhibited conserved motifs with reported Hsp70 proteins from other organisms (Clerico et al. 2019; Kiang and Tsokos 1998), indicating those motifs are important for maintaining the function of HmHsp70. Among all the Hsp70 proteins in *Saccharomyces cerevisiae,* HmHsp70 showed the highest sequence identity with SSC1. SSC1 is located in the mitochondria in *S. cerevisiae* (Craig and Jacobsen 1984; Craig et al. 1987). Besides, HmHsp70 does not contain the typical cytosolic compartment sequence, (GP (T/K) (V/I) EE (V/M) D), suggesting that *hmhsp70* is located in the mitochondria region.

HSPs, function as molecular chaperones, play critical roles in stress response to adverse environment (Kiang and Tsokos 1998; Kurahashi et al. 2014). In our study, the expression of *hmhsp70* increased under heat shock indicating that *hmhsp70* is involved in heat shock response in *H. marmoreus*. The result is consistent with the expression of Hsp70 genes in other mushrooms (Chen et al. 2017; Wang et al. 2018a; Zhang et al. 2016; Zou et al. 2018). The silencing of Hsp40 gene, *LeDnaJ*, in *L. edodes* defected its resistance to heat stress (Wang et al. 2018b), while the over-expression of *LeDnaJ* gene in *L. edodes* S606 conferred the strain better tolerance to heat stress (Wang et al. 2018a). Overexpression of an Hsp100 gene, *PsHsp100*, from *Pleurotus sajor*-*caju* complemented a thermotolerance defect in *hsp104* mutant *S. cerevisiae* (Lee et al. 2006). The expression increase of *hmHsp70* is not as high as *hsp70* genes in the cytosolic region (Wang et al. 2016). The same result was also reported in *S. cerevisiae,* that the increase of *SSC1* transcripts ranged from 1.5- to 4.0- fold after heat shock for 0.5 h (Craig et al. 1987). To investigate the role of *hmhsp70*, we introduced this gene into tobacco. In this study, the seedling plants were used as the experimental materials. The *hmhsp70* overexpression transgenic tobaccos exhibited higher heat tolerance in lethal temperature compared to WT lines. The results indicated that overexpression of *hmhsp70* gene in tobacco conferred enhanced tolerance to heat stress. Nwaka et al. (1996) reported that *SSC1* is necessary for recovery from heat shock in *S. cerevisiae*. In another study, increase expression of *SSC1* partially suppressed the cold sensitive growth defect of the *SSH1* mutant (Schilke et al. 1996). All the reports combined with our results indicate that *hmhsp70* may necessary for the heat tolerance and recovery in *H. marmoreus*.

HSPs play a vital role in heat stress response in edible mushroom, therefore, they have received a wide range of attention. Zhang et al. demonstrated that heat stress induces a significantly increased cytocolic Ca^2+^ level in *G. lucidum*. Cytosolic Ca^2+^ participates in heat shock signal transduction and the increased intracellular calcium triggers the accumulation of HSPs (Zhang et al. 2016). Liu et al. (2018) reported that heat stress induced a significant increase in the cytosolic reactive oxygen species concentration, which also participate in the regulation of HSP expression. The expressed HSPs function as molecular chaperones in blocking protein aggregation, dissolving the denatured proteins and helping damaged proteins to fold. Wang et al. reported that HSPs could regulate the IAA biosynthesis, and IAA accumulation could enhance thermotolerance of *L. edodes* (Wang et al. 2018a). These studies demonstrated that the metabolic network of HSPs synthesis and functions under heat stress are complicated. The diversity of HSPs is one of the reasons for this complexity. Werner-Washburne reported eight Hsp70 homologues in yeast, of which six are localized to the cytosolic region and two are located in mitochondria or endoplasmic reticulum (Werner-Washburne and Craig 1989) regions, respectively. Human also contains at least eight Hsp70 family proteins, which distribute in different regions the same as those in yeast (Daugaard et al. 2007). We search the Hsp70 proteins in the genome of *H. marmoreus* using BLAST software. Eight Hsp70s proteins were found in the genome of *H. marmoreus*. Other Hsp70 proteins in *H. marmoreus* may also participate in heat shock response, and the heat shock induced Hsp70 synthesis may have different sources. The mechanism of Hsp70 proteins involved in heat shock response in edible mushroom requires further study. This will provide a basis for improving the thermotolerance ability by genetic manipulation in the near future.

## Data Availability

The dataset supporting the conclusions of this article is included within the article. All data are fully available without restriction.

## Abbreviations

Hsp70s: 70-kD heat shock proteins
WT: wild type
HSPs: heat shock proteins
PDA: potato dextrose agar
MS: Murashige and Skoog
LB: Luria-Bertani
Kan: kanamycin
Hyg: hygromycin
qPCR: real-time quantitative PCR
